# A Special Ingredient (VtR) Containing Oligostilbenes Isolated from *Vitis thunbergii* Prevents Bone Loss in Ovariectomized Mice: *In Vitro* and *In Vivo* Study

**DOI:** 10.1155/2013/409421

**Published:** 2013-04-11

**Authors:** Yu-Ling Huang, Yen-Wenn Liu, Yu-Jou Huang, Wen-Fei Chiou

**Affiliations:** ^1^National Research Institute of Chinese Medicine, No. 155-1, Section 2, Li-Nong Street, Beitou District, Taipei 11221, Taiwan; ^2^Department of Cosmetic Science, Chang Gung University of Science and Technology, No. 261, Wen-hwa 1st road, Kwei-shan, Taoyuan 333, Taiwan; ^3^Department of Biotechnology, Hungkuang University, No. 1018, Section 6, Taiwan Boulevard, Shalu District, Taichung 43302, Taiwan

## Abstract

*Vitis thunbergii* is used in Taiwan as a botanical supplement for inflammatory bone diseases. This study aims to examine its direct effect on bone metabolism. Three-month-old female mice were randomly divided into ovariectomized control (OVX), sham operated (SHAM), and ovariectomy treated with either 17**β**-estradiol or a special ingredient (VtR) fractionated from an ethanol extract of *V. thunbergii* started two weeks after ovariectomy. VtR treatment for 8 weeks significantly ameliorated the deterioration of bone mineral density and reversed all the ovariectomy-induced changes in  **μ**-CT parameters. The antiosteoporotic effect of VtR accompanied decrease in serum levels of C-terminal telopeptides of type I collagen (CTx), interleukin-7, and ration of RANKL/osteoprotegerin (OPG) but rise in osteocalcin concentration. Sparse calcified microarchitecture and less alkaline-phosphatase- (ALP-) positive cells were observed at the femur and vertebral sites in OVX mice while VtR remarkably restored such variation. HPLC analysis showed (+)-vitisin-A, (−)-vitisin-B, and ampelopsin C predominated in VtR. Both (−)-vitisin B and ampelopsin C increased ALP activity and bone nodule formation in cultured osteoblasts. Instead of stimulating osteoblastogenesis, (+)-vitisin A dramatically repressed osteoclasts differentiation and bone resorption. The results suggested VtR composed of diverse components to reciprocally drive osteoblastogenesis and interdict osteoclastogenesis may serve as a potential botanic drug for osteoporosis therapy.

## 1. Introduction

Osteoporosis is characterized by skeletal degeneration with low bone mass and destruction of the microarchitecture of bone tissue which is attributed to various factors including inflammation and aging [1]. Women are more likely to develop osteoporosis than men due to the reduction in estrogen during menopause, leading to decreased bone-formation (mediated by osteoblasts) and increased bone-resorption (mediated by osteoclasts) activity. Currently available agents used to treat osteoporosis are major based on the inhibition of osteoclastic bone resorption to prevent further bone loss [2]. Drugs with anabolic effects have recently received much attention for osteoporosis therapy. These pharmacologic agents can ultimately stimulate new bone formation, enhance bone density, reduce bone fracture, and promote bone health.

**Figure 1:**
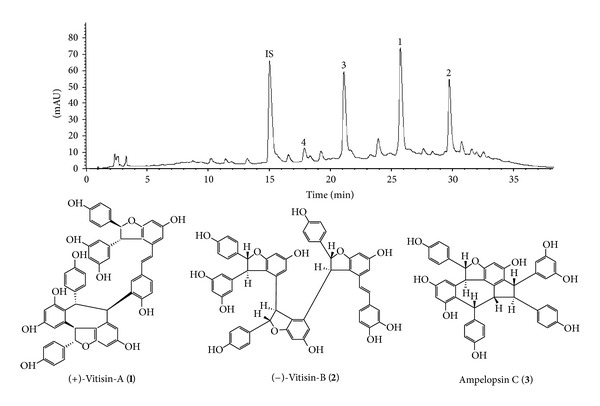
HPLC chromatograms of VtR and chemical structure of (+)-vitisin A (**1**), (−)-vitisin B (**2**), and ampelopsin C (**3**). Retention times for internal standard (4-hydroxy-3-methoxycinnamaldehyde, IS), ampelopsin C (**3**), (+)-vitisin A (**1**), and (−)-vitisin B (**2**) were 15.0, 21.1, 25.7, and 29.7 min, respectively. The traced component of peak 4 with a retention time of 17.9 min was resveratrol.

*Vitis thunbergii *Sieb. and Zucc. (*Vitis ficifolia *Bge. Vitaceae) is a famous herb traditionally used in Taiwan for treating diarrhea, jaundice, hepatitis, fracture, and injury [3]. Modern pharmacological studies reported that the crude extract of *V. thunbergii *displayed antihypertensive [4], antiatherogenic [5], antioxidative [6], and anti-inflammatory activities and was beneficial in lipopolysaccharide-induced arthritis [7]. Accumulating use experience in Taiwan revealed an alcoholic drench of the roots of *V. thunbergii* cures bone fractures and contusions and prevents bone loss, suggesting that this herb might stimulate new bone formation and accelerate fracture healing. However, so far there is no direct experimental evidence concerning the therapeutic benefit of *V. thunbergii* on bone metabolism, and which components contribute to this beneficial effect also remains unclear. Our previous study in investigation the chemical constituents of the roots of *V. thunbergii* demonstrated that (+)-vitisin A (**1**), (−)-vitisin B (**2**), and ampelopsin C (**3**) (Figure 1) were the three major compounds existed in the ethanol extract of this herb through HPLC analysis [8, 9]. Therefore, we initiated a project to prepare a partial purification concentrated with these three components (named as VtR) and evaluate its antiosteoporotic potential in ovariectomized mice. In this study, bone microarchitecture and bone mineral density (BMD) were assessed by microcomputed tomography (*μ*-CT), nondecalcified bone histology was assessed by Von Kossa or alkaline phosphatase (ALP) stain, and serum levels of C-terminal telopeptides of type I collagen (CTx), receptor activator of NF-*κ*B ligand (RANKL), osteoprotegerin (OPG), and interleukin-7 (IL-7) were measured by the standard colorimetric method and ELISA using commercial kits, respectively. Experiments were also conducted in primary cultured osteoblasts and osteoclasts to delineate the mechanisms involved. 

## 2. Materials and Methods

### 2.1. Animals

Specific pathogen-free (SPF) C57BL/6J female mice, 8 to 10 weeks of age, were obtained from the National Laboratory Animal Center (Taipei, Taiwan). Animals were housed at a constant temperature and fed with laboratory chow (PMI, Brentwood, MO, USA) and water ad libitum. The experimental protocol was approved by the Institutional Animal Care and Use Committee of the National Research Institute of Chinese Medicine (IACUC Approval Number: 97-A-09).

### 2.2. Preparation of VtR

The roots of *V. thunbergii* were purchased from a plantation in Tainan, Taiwan, in July, 2010 and identified by Dr. I-Jung Lee of the Herbarium of National Research Institute of Chinese Medicine, China. A voucher specimen (NHP-00845) is deposited at this herbarium. The air-dried roots of *V. thunbergii* (600 g) were heated under reflux with 95% EtOH/H_2_O = 1 : 1 (3 × 2.5 L, 40 min each). After filtration, the filtrate was concentrated under vacuum to provide an EtOH/H_2_O extract (Vt, 104.2 g). Dissolving 43.3 g of Vt in 100 mL EtOH yield 20.8 g EtOH-soluble extract was then subjected to a Sephadex LH-20 column (7 × 45 cm, GE Healthcare) eluting with EtOH to collect 41 fractions (250 mL each). Combining fractions 23–45 afforded the test sample named as VtR concentrated with (+)-vitisin A, (−)-vitisin B, and ampelopsin C.

### 2.3. HPLC Analysis of VtR

HPLC was conducted on an Agilent 1100 series equipped with a G1311A QuatPump, G1379A degasser, G1315B photodiode array detector, and a 1200 series G1329A ALS. The separations were performed on a Cosmosil 5C18-AR-II column (5 *μ*m, 250 × 4.6 mm i.d.) with a mobile phase of acetonitrile/water mix using a linear gradient, starting from 10% acetonitrile, increasing the ratio to 20% in 5 min, then to 50% at 35 min, and finally reaching 100% at 40 min. The flow rate was 1.0 mL/min. The UV detection wavelength was set at 280 nm [8]. HPLC profile of VtR showed that (+)-vitisin A, (−)-vitisin B and ampelopsin C were the major constituents (Figure 1).

4-Hydroxy-3-methoxy-cinnamaldehyde was used as an internal standard (IS) with a purity of 99.0%. Retention times (RT) for IS, ampelopsin C, (+)-vitisin A, and (−)-vitisin B were 15.0, 21.1, 25.7, and 29.7 min, respectively. The traced component of peak 4 with a retention time of 17.9 min was resveratrol. The isolation of (+)-vitisin A, (−)-vitisin B, and ampelopsin C was described previously with the purities of 99.4, 97.8, and 99.1%, respectively [8, 9]. Resveratrol was purchased from Merk KGaA (Darmstadt, Germany) with a purity of 98.0%.

### 2.4. Model Establishment and Treatment Protocol

**Figure 2:**
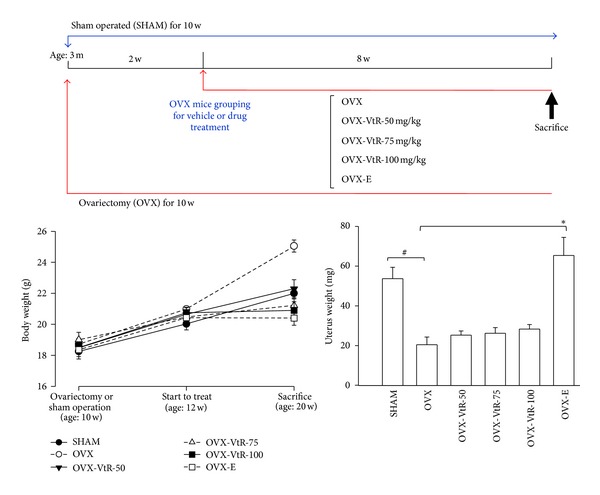
The experimental design and effects of VtR on body weight and uterine weight in sham-operated mice (SHAM), ovariectomized mice administered with vehicle (OVX), different dosages (50, 75, and 100 mg/kg) of VtR (OVX-VtR-50, -VtR-75, and -VtR-100), or 17*β*-estradiol (OVX-E) for 8 weeks. Values are mean (*n* = 8–10) with their standard error of mean (S.E.M.) represented by vertical bars. ^#^ *P* < 0.01, significantly different from the SHAM group; *P < 0.01, significantly different from the OVX group.

Sixty female C57BL/6J mice (8 weeks old, from National Laboratory Animal Center, Taipei, Taiwan) were housed in an environmentally controlled laboratory upon arrival and acclimatized for 7 days to a rodent chow diet and distilled water ad libitum. After that, mice were fed a commercial pelleted soy-free diet (product D10001; Research Diets, New Brunswick, NJ, USA) throughout the experiment. The commercial diet consisted of ~20% protein, 5.5% fat, and 66% carbohydrate. At 10 weeks of age, the animals were either bilateral ovariectomized or sham operated (SHAM). Two weeks after surgery, the ovariectomized mice were divided into five groups: oral treated with vehicle (OVX), 50 mg/kg of VtR (OVX-VtR-50), 75 mg/kg of VtR (OVX-VtR-75), 100 mg/kg of VtR (OVX-VtR-100), or subcutaneous injection of 17*β*-estradiol (OVX-E, 50 *μ*g/kg/day) for 8 weeks (*n* = 10/each group). Vehicle (corn oil) and VtR were administered orally through a custom-made stomach tube. All mice were sacrificed at the age of 20 weeks. The experimental procedure is summarized in Figure 2. The body weight of each animal was measured weekly, and the dosage of drug or vehicle administered was calculated based on the most recent body weight measurement. After 8 weeks of drug treatment, the experimental mice were fasted overnight, anesthetized, and then blood was sampled from the heart. Serum was isolated from the blood samples by centrifugation at 3000 ×g, 4°C, for 5 min and stored at −70°C prior to biochemical measurement. The lumbar vertebral bodies and femora were immediately dissected free of soft tissues. The 4th lumbar vertebra (LV4) and the left femur were collected from each animal for histomorphometry. The second and third lumbar vertebra (LV2-LV3) and right femur were collected for *μ*-CT to measure the microstructural parameters and bone mineral densitometry (BMD). The uterine, heart, liver, spleen, lung, and kidney were removed from each mouse and immediately weighed.

### 2.5. Analysis of Bone Microarchitecture by Microcomputed Tomography (*μ*-CT)

The cortical and trabecular bone microstructure and a 3D image of the distal femoral metaphysis were analyzed using the SkyScan1076 *in vivo μ*-CT scanner (SkyScan, Aartselaar, Belgium) in the region of 0.6–2.1 mm from the growth plate. The X-ray source was set at a voltage of 50 kV and a current of 140 *μ*A and filtered with a 0.5 mm aluminum filter. The scanning angular rotation was 180° with an angular step of 0.8°. The voxel size was isotropic and fixed at 9 *μ*m. The trabecular bone parameters were calculated using the SkyScan software CTAn (SkyScan). Morphometric indices of the trabecular bone region were determined from the microtomographic data sets using direct 3D morphometry. The structural parameters including bone volume (BV), total volume (TV), bone surface density (BS/TV), trabecular number (Tb.N), trabecular separation (Tb.Sp), and trabecular thickness (Tb.Th) were calculated. The volumetric cortical bone mineral density (BMD) was also determined by *μ*-CT scanning followed the protocol described by Peng et al. [10]. The operator conducting the scan analysis was blinded to the treatments associated with the specimen.

### 2.6. Histomorphometric Analysis

Specimens including the left femurs and the lumbar vertebral (LV4) bodies were fixed in 10% neutral buffered formalin, dehydrated in increasing gradients of alcohol, and embedded in methylmethacrylate resin (Eastman Organic Chemicals, Rochester, NY, USA). For von Kossa staining, the undecalcified parasagittal sections of lumbar vertebral and ground sections parallel to the long axis of the femur were obtained at a final thickness of 4–6 *μ*m. Resin sections were incubated with an aqueous solution of 5% silver nitrate (Wako Pure Chemical Industries, Tokyo, Japan) for 60 min at room temperature under sunlight until they took on a dark brown color. Following a distilled water rinse, sections were incubated with a 5% sodium thiosulfate solution (Wako Pure Chemical Industries) for 5 min followed by image acquisition. The sections of interest were further performed for bone alkaline phosphatase (ALP) staining to determine osteoblast activity. Tissue ALP enzyme levels were measured by ALP staining kit (Sigma-Aldrich, St. Loius, MO) according to the manufacturers' instructions.

### 2.7. Serum Markers of Bone Metabolism and Interleukin-7 (IL-7)

Mice were fasted overnight before morning blood collection. Serum osteocalcin (OCN) level and the sensitive biomarkers of bone formation were estimated by an osteocalcin EIA kit (Biomedical Technologies, Stoughton, MA, USA) according to the manufacturers' instructions. The serum CTx concentration was measured in blood using an ELISA RatLaps kit (Nordic Bioscience Diagnostics A/S, Herlev, Denmark). RANKL and OPG were determined using ELISA from R&D Systems (Minneapolis, MN, USA). To dose serum IL-7 levels, we performed an ELISA assay, according to the manufacturer's instructions (R&D Systems, Abingdon, UK). The kit sensitivity was 0.27–8.7 pg/mL.

### 2.8. Cell Culture for Measuring Osteoblasts Differentiation and Proliferation

Primary mouse bone marrow cells (BMCs) were cultured as described previously [11, 12]. Briefly, BMCs were obtained from the femoral bone of SPF-grade 5-week-old C57BL/6J female mice. The BMCs were collected by flushing the diaphysis with serum free *α*-minimum essential medium (*α*-MEM, Invitrogen, Carlsbad, CA, USA) through a 23-gauge needle. After flushing, the BMCs were filtered through a no. 53 sterile nylon mesh to obtain a single cell suspension. The cells were cultured in osteogenic induction medium (*α*-MEM medium supplemented with 15% fetal calf serum (FCS, Gibco), 50 *μ*g/mL ascorbic acid, 10 mM sodium *β*-glycerophosphate, and 10 nM dexamethasone (Sigma-Aldrich)).

For the ALP activity assay, the well-known marker of osteoblast differentiation, primary BMCs were seeded in a 96-well microplate at 2.5 × 10^5^/well and cultured in osteogenic induction medium. After two days, half of the medium was changed and the cells were incubated with tested compounds at the indicated concentrations for 4 days. ALP activity was assessed by a colorimetric assay measuring the conversion of *p*-nitrophenyl phosphate to *p*-nitrophenyl in cell lysates as described previously [13, 14]. Cell growth was measured by BrdU incorporation using the cell proliferation ELISA Biotrack kit (Amersham Biosciences, Piscataway, NJ, USA) and cytotoxicity was determined by 3-(4,5-dimethylthiazol-2-yl)-2,5-diphenyl tetrazolium bromide (MTT) assay.

### 2.9. Nodule Formation Assay

For the nodule formation assay, primary BMCs were seeded in a 6-well microplate at 4 × 10^6^/well and cultured in osteogenic induction medium. After two days, half of the medium was changed and the cells were incubated with tested compounds at the indicated concentrations. The culture medium was renewed every 4 days. After 14 days, the mineralization of BMCs was analyzed as described previously [13–15]. In brief, cells were fixed with 10% formalin for 30 minutes at 37°C. The formalin was removed, and the cells rinsed with sterilized water three times. Next, 2% Alizarin Red S solution, which reacts with calcium, was added to the wells and the cells were incubated at 37°C for 10 minutes. The Alizarin Red S solution was removed, and the cells were washed with water and dried in air. Stained cultures were photographed.

### 2.10. Primary Bone Marrow Cell Culture and Osteoclast Differentiation

BMCs were cultured with *α*-MEM medium supplemented with 15% FCS (Gibco) and 10 ng/mL macrophage-colony stimulating factor (M-CSF; R&D Systems, Minneapolis, MN, USA) for 2 days, and they were used as bone marrow macrophages (BMMs). BMMs were further cultured for 4 days in differentiation medium (*α*-MEM medium supplemented with 15% FCS (Gibco), 50 ng/mL RANKL (R&D Systems), and 10 ng/mL M-CSF). After differentiation, adherent cells were stained for tartrate-resistant acid phosphatase (TRAP), an osteoclast marker enzyme, using a TRAP staining kit (Sigma-Aldrich) [12]. Briefly, cells were washed with PBS and treated with a fixation solution at room temperature for 5 min. They were then washed with distilled water and treated with a TRAP reagent at 37°C for 20–60 min. After washing with distilled water, the cells were observed under a microscope. Cells possessing three or more nuclei were identified as multinucleated cells (MNCs). TRAP-positive MNCs (TRAP^+^MNCs) were counted as osteoclast-like cells under the light microscope [12]. Cell growth was measured by BrdU incorporation assay and cytotoxicity was determined by MTT assay.

### 2.11. Functional Bone-Resorption Assay

The resorptive function of mature MNCs derived from RANKL-differentiated BMMs was analyzed on Osteologic Plates (BD BioCoat Osteologic Bone Cell Culture System, BD Biosciences, San Jose, CA, USA). Briefly, mouse BMMs were seeded on bone slices and treated with RANKL (50 ng/mL) and M-CSF (10 ng/mL) to induce differentiation into mature osteoclasts for 6 days. After mature MNCs had formed, the cells were treated with or without (+)-vitisin A (2.5, 5 and 10 *μ*M) in the presence of M-CSF and RANKL for a further 48 h. Cells were then removed in 1 N NaOH for 20 min, the slices were washed twice with PBS, and the resorption pits were visualized by staining with Mayer's hematoxylin (Sigma) for 30 s. Finally, bovine bone slices were transferred onto glass slides, mounted with glycerol, covered with glass cover slips, and observed under a light microscope. The resorption area was observed under a light microscope and analyzed by Image-Pro Plus [12].

### 2.12. Statistical Analysis

Data are expressed as mean ± S.E.M. The statistical significance was evaluated by one-way analysis of variance (ANOVA) followed by Dunnett's test. A level of *P* < 0.05 was considered statistically significant.

## 3. Results

### 3.1. VtR Prevents Bone Lose in OVX-Induced Osteoporotic Mice

#### 3.1.1. Weight Gain and Uterus Index

The changes in the body weights of the mice in each group are shown in Figure 2 (bottom). Six groups of mice had a similar initial mean body weight. Although there was a trend for greater weight gain in the five ovariectomized groups measured at the age of 12 weeks (before treatment), it did not reach statistical significance. As expected, the body weight of the OVX group (open circle with short dash) continued to be significantly higher than the SHAM group (filled circle with solid line) throughout the study. 17*β*-Estradiol (E) (open square with solid line) completely prevented the increase in body weight associated with estrogen deficiency and returned the body weight to the level maintained by the SHAM group 8 weeks after treatment. Treatment with different doses of VtR for 8 weeks also significantly prevented the body weight increase compared to that of the OVX group.

We also measured the uterine weight (as a measure of the completeness of OVX and response to therapy) of six groups of mice. As shown in Figure 2 (bottom), ovariectomy for 10 weeks caused significant atrophy of uterine tissue compared to the SHAM group, indicating the success of the surgical procedure. Administration of 17*β*-estradiol at two weeks after ovariectomy (OVX-E group) displayed a clear uterotrophic effect observed by completely preventing the decrease in uterine weight for the 8-week treatment period. On the other hand, those of the VtR-treated OVX mice were similar to that of ovariectomized mice treated by vehicle only (OVX), suggesting that VtR by itself had no effect on uterine weight. The tissue weight of the heart, liver, spleen, lung, and kidney did not significantly differ in each group (data not shown).

#### 3.1.2. Bone Mineral Density (BMD)

**Figure 3:**
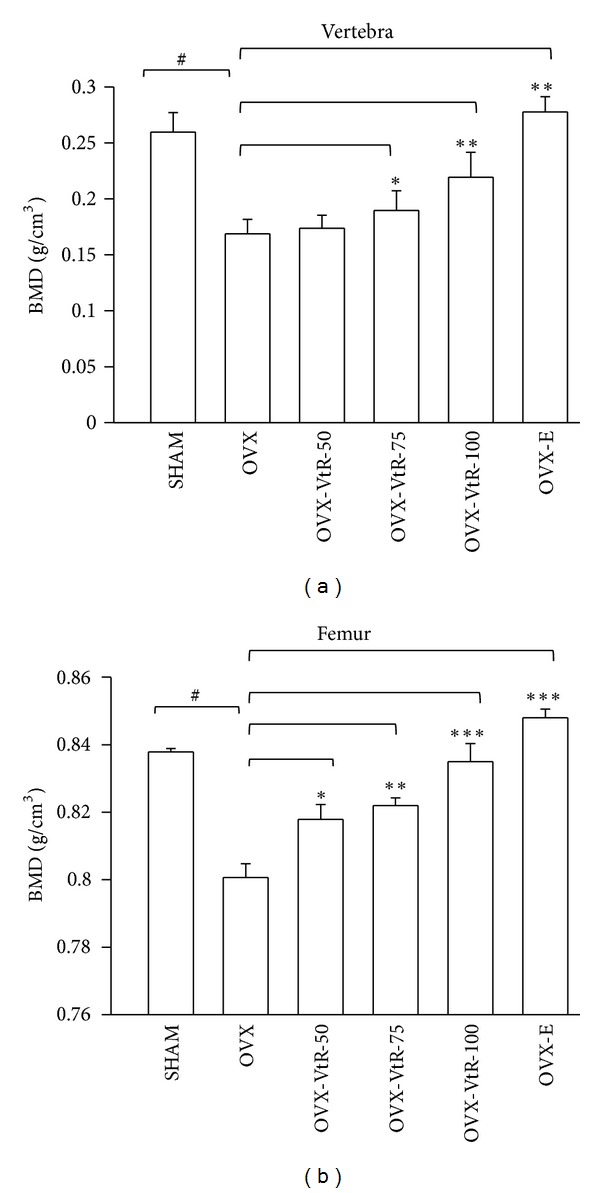
Bone mineral density (BMD) in vertebra and femur of sham-operated mice (SHAM), ovariectomized mice administered with vehicle (OVX), different dosages (50, 75, and 100 mg/kg) of VtR (OVX-VtR-50, -VtR-75, and -VtR-100), or 17*β*-estradiol (OVX-E) for 8 weeks. Values are mean (*n* = 10) with their standard error of mean (S.E.M.) represented by vertical bars. ^#^ *P* < 0.01, significantly different from the SHAM group; *P < 0.05, **P < 0.01, and ***P < 0.001, significantly different from the OVX group.

Osteopenia was evident at both the lumbar vertebrae and femur sites in OVX mice compared with age matched SHAM control at the end of the study. The results showed that the BMD of lumbar vertebrae were significantly decreased by 38% in OVX as compared with the SHAM control (Figure 3). Administration of VtR for 8 weeks tended to have a dose-dependent antiosteoporotic effect; that is, treatment with 75 and 100 mg/kg of VtR significantly prevented vertebral BMD loss induced by ovariectomy. In comparison with the OVX group, the OVX-VtR-75 and OVX-VtR-100 groups demonstrated a 13% and 26% greater BMD than OVX, respectively. Further, the BMD of femur was also significantly decreased by 54% in the OVX group as compared with the age matched SHAM. Treatment with VtR resulted in a dose-dependent reversal of bone loss at femur site induced by ovariectomy; that is, the decreased BMD in OVX mice was prevented by approximately 22, 30, and 49% by 50, 75, and 100 mg/kg VtR treatment (*P* < 0.05), respectively. The aforementioned results indicate that the BMD in VtR-treated groups was significantly higher than that of the OVX groups.

#### 3.1.3. *μ*-CT Evaluation

**Figure 4:**
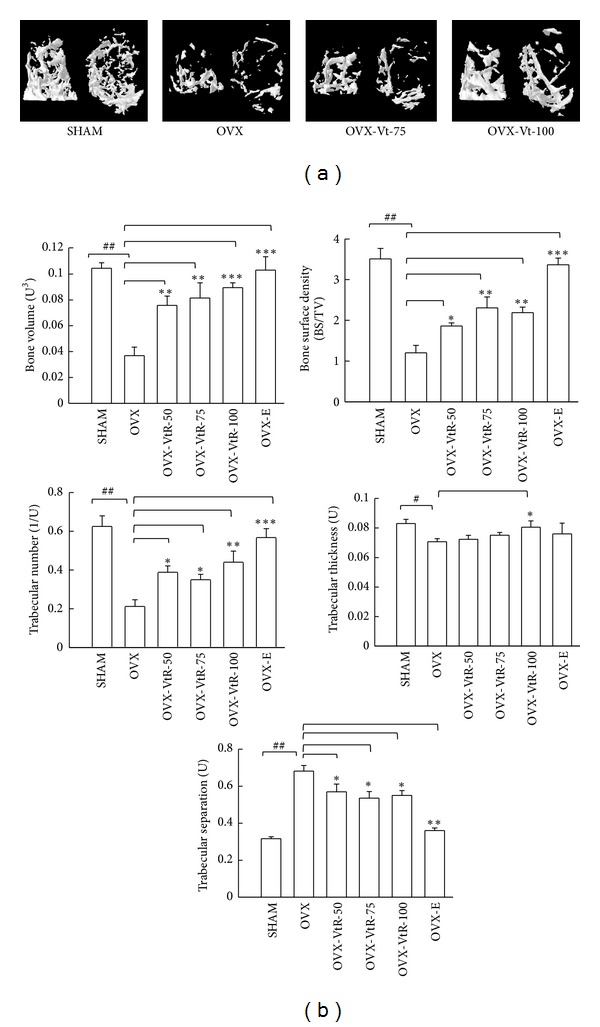
Representative *μ*-CT images and selective *μ*-CT parameters of the distal femurs. (a) 3D tomographic rendering of distal femoral metaphysis trabecular bone reveals the complexity of the bone structure. The image on the left shows the side view, and the image on the right shows the top view. The abbreviations appearing here are the same as below. (b) Changes in femora microstructural parameters measured by *μ*-CT in sham-operated mice (SHAM), ovariectomized mice administered with vehicle (OVX), different dosages (50, 75, and 100 mg/kg) of VtR (OVX-VtR-50, -VtR-75, and -VtR-100), or 17*β*-estradiol (OVX-E) for 8 weeks. Values are mean (*n* = 8–10) with their standard error of mean (S.E.M.) represented by vertical bars. ^#^ *P* < 0.05 and ^##^ *P* < 0.01, significantly different from the SHAM group; *P < 0.05, **P < 0.01, and ***P < 0.001, significantly different from the OVX group.

Figure 4 showed ovariectomy-induced significant changes in the femora microstructure and parameters measured by *μ*-CT. Compared with SHAM rats, OVX for 10 weeks significantly reduced bone volume (BV) by 63%, bone surface density (BS/TV) by 71%, trabecular number (Tb.N) by 75%, and trabecular thickness (Tb.Th) by 14% and increased trabecular separation (Tb.Sp) by 107% (Figure 4(b)). All VtR-treated groups evinced significant improvement of the decreasing structural factors induced by OVX. In particular, treatment with the middle (75 mg/kg) and high (100 mg/kg) doses of VtR produced better results than the low (50 mg/kg) dose of VtR for most of the parameters, recovering the reduced BV from 33 to 76 and 85%, BS/TV from 29 to 69 and 58%, Tb.N from 34 to 51 and 63%, and Tb.Th from 81 to 85 and 93%, while preventing the increase of Tb.Sp from 113 to 54 and 56%, respectively. These increments in trabecular bone parameters were readily observable in the *μ*-CT images of the femur. As shown in Figure 4(a), all VtR-treated groups formed a tight and dense structure at the proximal region of the femur compared with that of the OVX group. Compared to the OVX group, subcutaneous injection of 50 *μ*g/kg/day of 17*β*-estradiol (OVX-E) largely reversed the above mentioned changes.

#### 3.1.4. Histological Analysis of Trabecular Bone

**Figure 5:**
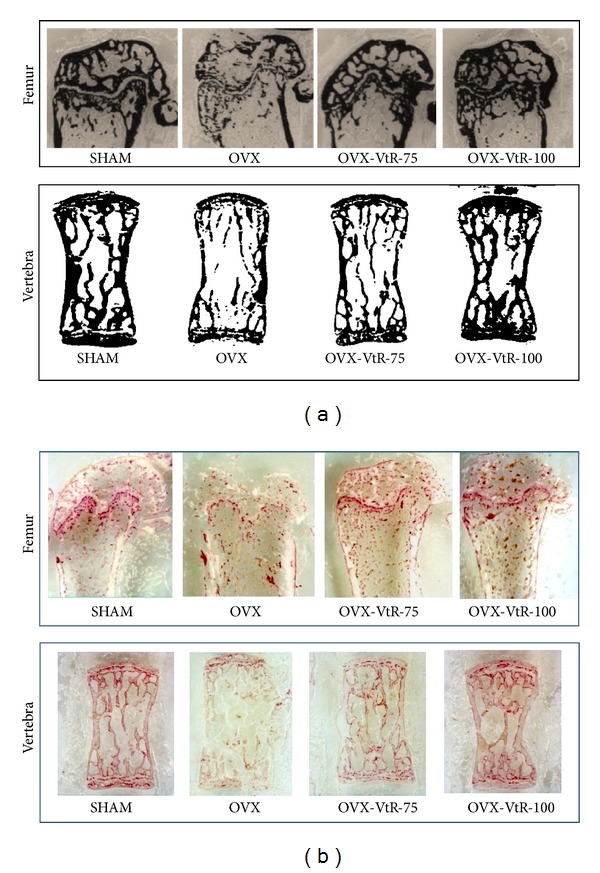
Histochemical staining for (a) calcium deposition and (b) alkaline phosphatase (ALP) in the femur and vertebra of sham-operated mice (SHAM), ovariectomized mice administered with vehicle (OVX), different dosages (75 and 100 mg/kg) of VtR (OVX-VtR-75 and OVX-VtR-100), or 17*β*-estradiol (OVX-E) for 8 weeks.

The extent of bone loss following ovariectomy was also evident after the histological analysis using Von Kossa staining. As shown in Figure 5(a), the trabecula region of the OVX group exhibited small, thin, and sparse morphology compared with that of the SHAM group at femur sites. The distal bone of the femur in the 75 and 100 mg/kg VtR-treated groups demonstrated thickened, dense architecture consistent with the *μ*-CT images. The mineral deposition in vertebral cancellous bone was also visualized by Von Kossa staining. The results revealed that decreased mineral deposition was observed in OVX mice as compared with the SHAM group and bone from the OVX-VtR-100 group displayed a more compact structure than bone from the OVX group. ALP stained intensity and proportion represented the osteoblasts activity. Many ALP-positive osteoblasts were present in epiphyseal line of distal femur. A decreased ALP-positive staining was observed at the femur sites in OVX mice compared with the SHAM (Figure 5(b)). The femur bone in 75 and 100 mg/kg VtR-treated groups demonstrated more intensive stain, which was consistent with the *μ*-CT images. A similar phenomenon appeared at the vertebra site.

#### 3.1.5. Serum Biochemical Markers

Ten weeks after OVX, the serum biochemical markers of bone turnover OCN, had increased in OVX mice compared with their SHAM controls. As shown in Table 1, the OVX mice showed on average 39% higher OCN levels. OVX mice administered with 75 and 100 mg/kg VtR resulted in further increases in serum levels of OCN as compared with OVX without treatment (Table 1). However, OVX mice treated with 17*β*-estradiol (E) led to a significant decrease in OCN level as compared with OVX without treatment. Consistent with the results on bone mass and histomorphometric data, we found that the serum level of bone-resorption marker CTx had increased at ten weeks after OVX. Serum CTx in the OVX group was about 1.3-fold the corresponding SHAM group value. A low dose (50 mg/kg) of VtR tended to decrease serum CTx content, but no significant difference was observed between the OVX and OVX-VtR-50 groups. Nevertheless, treatment with 75 and 100 mg/kg of VtR showed a significant decrease compared to the OVX group.

Osteoblasts can secrete OPG to protect the skeleton from excessive bone resorption by binding to RANKL and preventing it from binding to its receptor, receptor activator of nuclear factor-*κ*B (RANK). Thus, RANKL/OPG ratio is an important determinant of bone mass and skeletal integrity. Ten weeks after ovariectomy, the serum RANKL/OPG ratio, a bone-resorption marker, had dramatically increased in the OVX group compared with the SHAM group. VtR at higher doses (75 or 100 mg/kg/day) significantly reduced serum RANKL/OPG ratio compared to the OVX group. These effects appeared to be dose dependent. 17*β*-Estradiol (E) treatment had a comparable effect as the highest dose of VtR in reducing the bone-resorption marker. The IL-7 serum value in the SHAM control group was 0.32 ± 0.05 pg/mL. Significant higher levels of IL-7 were found in mice post-ovariectomy for 10 weeks. Both VtR and 17*β*-estradiol (E) treatment significantly decreased the serum IL-7 production in OVX mice.

### 3.2. (−)-Vitisin-B and Ampelopsin C Promote Osteoblastogenesis

**Figure 6:**
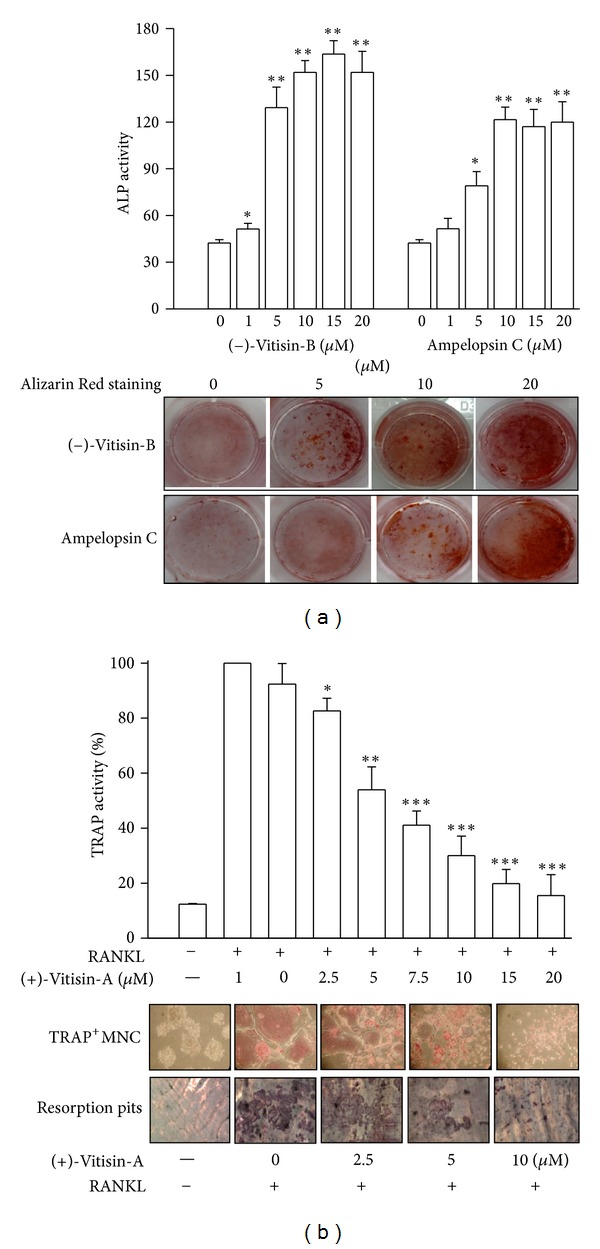
Effects of oligostilbenes isolated from *Vitis thunbergii* on the differentiation and function of primary cultured osteoblasts and osteoclasts, respectively. (a) (−)-Vitisin B and ampelopsin C concentration-dependently stimulated the increases in ALP activity and mineral matrix deposition (Alizarin Red staining) in osteoblasts, respectively. The cells were incubated with the tested drug for 7 or 14 days as described in Materials and Methods. Each value is the mean ± S.E.M. of six independent experiments, each in triplicate. *P < 0.05 and **P < 0.01, compared with the control group without the tested drug treatment. (b) (+)-Vitisin A concentration-dependently repressed RANKL-induced osteoclast differentiation, TRAP^+^MNCs formation, and bone resorption. Mouse BMM cells were cultured for 4 or 8 days in the presence of M-CSF and RANKL, as described in Materials and Methods.. Results are presented as means ± S.E.M. of four independent experiments. *P < 0.05, **P < 0.01, and ***P < 0.001, as compared with RANKL.

Based on the above finding that VtR ameliorates experimental osteoporosis in ovariectomized mice, a study was further conducted to analyze the effects of three major oligostilbenes existed in VtR on cell proliferation and differentiation/mineralization in primary mouse osteoblasts and compared with resveratrol. None of the tested compounds significantly affected osteoblast growth at a concentration of 20 *μ*M over a 4-day culture period (data not shown). Measuring the osteogenic potential of each compound at a concentration of 20 *μ*M, 1.6-, 3.4-, and 2.7-fold increases in ALP activity were observed by (+)-vitisin A, (−)-vitisin B, and ampelopsin C treatment, respectively, as compared to the control (defined as 1). Resveratrol failed to display a notable osteogenic effect under the same condition and even reduced cell viability by about 30% (Table 2). The concentration-response relationship of (−)-vitisin-B and ampelopsin C was further constructed.  Figure 6(a) showed that (−)-vitisin-B concentration-dependently increased ALP activity, beginning at 1 *μ*M and reaching a maximum between 10 and 20 *μ*M. Ten *μ*M (−)-vitisin-B increased ALP activity to 3.5 times the control. Ampelopsin C also increased ALP activity to 1.1-, 1.9-, and 2.8-fold as compared with the control. A maximal response was observed at concentrations ranging from 10 to 20 *μ*M. To evaluate whether (−)-vitisin-B or ampelopsin C enhanced bone mineralization, we used Alizarin Red staining to visualize nodule formation in bone marrow cultures (Figure 6(a), bottom). The proportional area of Alizarin-Red-positive staining in the (−)-vitisin-B- or ampelopsin C-treated group was larger than that in the untreated control group in a dose-dependent manner. The addition of (−)-vitisin-B and ampelopsin C for a 14-day cultured period did not display detectable cytotoxicity (data not shown).

### 3.3. (+)-Vitisin-A Inhibits Osteoclastogenesis and the following Bone Resorption

None of the tested compounds significantly affected osteoclast growth at a concentration of 20 *μ*M over a 4-day culture period (data not shown). We further studied their effects on osteoclastogenesis by determining the osteoclast differentiation marker TRAP after the stimulation of BMMs with recombinant RANKL in the presence of M-CSF. At a concentration of 20 *μ*M, (+)-vitisin A displayed the most potent effect in suppressing osteoclast differentiation, evidenced by decreasing TRAP activity by almost 95% (Table 2). The resident oligostilbenes exhibited undetectable to mild inhibition. Resveratrol also showed an inhibitory effect on osteoclast activity; this was associated with significant cytotoxic effects (Table 2). The concentration-response relationship of (+)-vitisin A (1–20 *μ*M) on TRAP activity was further constructed in Figure 6(b) (top). (+)-Vitisin-A at a concentration of 2.5 *μ*M gradually reduced the RANKL-induced TRAP activity by 20% in BMMs. At 5, 10, and 20 *μ*M, it robustly reduced the activity by 62, 87, and 94%, respectively. The inhibition of TRAP activity was not due to cytotoxicity because MTT analysis revealed that (+)-vitisin-A at a concentration up to 20 *μ*M did not display notable harmful effects on cell viability (Table 2). Meanwhile, many TRAP-positive multinucleated cells (TRAP^+^MNC) were formed in the culture within 4 days in response to RANKL (Figure 6(b), middle). (+)-Vitisin-A added to the culture for the entire culture period concentration-dependently inhibited TRAP^+^MNC formation induced by RANKL.

In the next experiment, the effect of (+)-vitisin-A on the bone-resorbing activity of mature osteoclasts was evaluated. After stimulation with RANKL/M-CSF for the entire 8 days, many resorption pits were formed on the bone slices in the absence of (+)-vitisin A. The pit formation area was concentration-dependently diminished by the addition of (+)-vitisin-A (2.5, 5, and 10 *μ*M) into mature MNCs (Figure 6(b), bottom). These results indicated, (+)-vitisin-A was able to inhibit bone resorption except for the disruption of osteoclast differentiation. The inhibitory effects of (+)-vitisin-A on pit forming were not due to cytotoxic effects because a similar survival rate was observed in cultures before and after (+)-vitisin-A treatment for a 2-day incubation of mature MNCs (data not shown).

## 4. Discussion

*Vitis thunbergii* is an endemic plant widely used for many years in Taiwan to strengthen the bone and encourage the bone healing. Wang et al. reported oligostilbenes isolated from *V. thunbergii *ameliorated lipopolysaccharide-induced knee arthritis by reducing serum PGE_2_ concentrations [7]. Although *V. thunbergii* has been considered as therapeutic agent to treat bone diseases, whether this herb modulates bone metabolism remained unknown. In the present study, we used an ovariectomized (OVX) mouse model to mimic the osteoporosis seen in postmenopausal women and evaluated the effect of a special processed ingredient (VtR) fractionated from the ethanol extract of *V. thunbergii *on bone metabolism.

In OVX animals, like in postmenopausal women, bone loss induced by ovarian deficiency mainly results from trabecular bone loss [16]. As expected, ovariectomy greatly reduced BMD, Tb.N, and Tb.Th while seriously elevated Tb.Sp in mice (Figures 3 and 4). Administration of VtR for 8 weeks has a therapeutic value in reversing bone loss evidenced by a significant rise in the BMD of the femur and vertebra, obvious increasing in Tb.N, and decreasing in the Tb.Sp of distal femur. The loss of bone mass and the deterioration of bone microstructure have been linked to an imbalance between bone formation and bone resorption [17]. Biochemical markers of bone turnover have been widely used to measure the status of bone remodeling. Type I collagen accounts for more than 90% of the organic matrix of bone and is synthesized primarily in bone. During renewal of the skeleton, bone matrix is degraded and consequently fragments of type I collagen are released into circulation. C-Terminal telopeptide of type I collagen (CTx) is a bone related degradation product from type I collagen and also a well known marker for bone resorption. Osteocalcin (OCN), one of the very few molecules exclusively produced by osteoblasts, is a widely used marker for bone formation which increases in osteoporosis of OVX rats and postmenopausal women [18, 19]. The present results showed that the serum concentrations of OCN and CTx in OVX mice were significantly higher than those in SHAM mice, indicating the increase of a bone turnover rate after OVX. Our results agree with those from Wronski et al., which demonstrated bone remodeling is accelerated after the cessation of ovarian function [20]. Further increase in OCN levels was observed in VtR treated group but not in 17*β*-estradiol (E) treated group, as compared to OVX without treatment. On the other hand, serum CTx in OVX mice were dose-dependently restored near to the levels in SHAM mice after VtR treatment for 8 weeks (Table 1). Collectively, these findings provided a proof of an important role for VtR in preventing bone lose through both bone-resorption inhibition and bone-formation enhancement.

 Estrogen deficiency causes bone loss mainly as a consequence of excessive bone resorption by osteoclasts. Because estrogen deficiency is accompanied by accelerated osteoclastogenesis, regulation of osteoclastogenesis is central to understanding the pathogenesis and treatment of estrogen deficiency-induced osteoporosis. Accumulated evidence has suggested osteoclast differentiation is principally stimulated by an increase in the ratio of RANKL to OPG in bone. OPG and RANKL are mainly produced by osteoblasts but also various other tissues. The binding of RANKL to RANK, its natural receptor which is expressed by osteoclasts, accelerates bone resorption. OPG acts as a decoy receptor and prevents the interaction of RANKL with RANK, therefore leading to decreased activity, survival, and proliferation of osteoclasts. The measurement of RANKL and OPG in serum had led to intensive research in the field of metabolic bone disease [21]. Preclinical data clearly indicated high RANKL and low OPG levels that is, a suppressed OPG/RANKL ratio is associated with high bone turnover and bone loss, though clinical evidence for this is missing. These findings were underscored by a negative correlation of RANKL and BMD at the lumbar spine [22]. Current therapies used to prevent or treat metabolic bone diseases are thought to act, at least in part, through modifying the RANKL/OPG dipole. The results obtained from Table 1 indicate VtR significantly down-regulated the ratio of RANKL/OPG. These findings support the assumption that the beneficial effect of VtR on bone formation might act, at least in part, through modulating the balance of RANKL/OPG, hence blocking bone resorption.

A hypothesis for bone loss in osteoporosis is that estrogen deficiency induces an unregulated chronic inflammatory process by increasing the local production of various osteoclastogenic cytokines and growth factors by cells located within the bone microenvironment [23, 24]. This is why proinflammatory cytokines are elevated in postmenopausal women and estrogen replacement therapy is able to suppress the production of pro-inflammatory cytokines [25]. One of the cytokines responsible for the enhanced osteoclastogenesis in states of estrogen deficiency is prostaglandin E_2_ (PGE_2_), a factor which induces osteoclast formation by upregulating stromal cell production of RANKL and M-CSF, and by augmenting the responsiveness of osteoclast precursors to RANKL [26, 27]. *Vitis* species are rich in phytophenols and have been used for centuries as therapeutic agents for treating inflammatory diseases, with its NO and PGE_2_ inhibitory properties [28, 29]. We previously reported that oligostilbenes isolated from *V. thunbergii* display potent antioxidant and anti-inflammatory activities [9]. Among the oligostilbenes tested, (+)-vitisin A, (−)-vitisin B, and ampelopsin C were the most potent three. Wang et al. also found that (−)-vitisin B, ampelopsin C, and (+)-vitisin A displayed notable anti-inflammatory activity to repress PGE_2_ production in human chondrocytes cultures stimulated by LPS [7]. Therefore, the ameliorating effect of VtR on bone resorption might be probably associated, at least in part, with its anti-inflammatory activity. Another cytokine relevant for osteoclast formation is IL-7, a powerful lymphopoietic cytokine that has previously been recognized as a potent inducer of bone destruction *in vivo* [19, 30]. How IL-7 leads to bone loss is controversial, and its mechanisms of action are only now beginning to be elucidated. IL-7 regulates multiple stages of T cell development and homeostasis. Indeed, IL-7 has been reported to induce production of RANKL by human T cells [31], and injection of IL-7 into mice *in vivo* induces bone destruction by eliciting the secretion by T cells of the key osteoclastogenic cytokines RANKL [32, 33]. In addition, level of IL-7 is significantly elevated following ovariectomy and neutralization of IL-7 by administration of its specific antibody prevents bone resorption induced by OVX [34, 35]. Remarkable progress had been made in elucidating the cross-talk between the immune system and bone and perhaps estrogen therapy prevents bone loss by regulating T cell function and immune cell bone interactions [36]. The present results also confirmed the increase of IL-7 production in OVX mice as previously reported. Further, OVX mice treated with 17*β*-estradiol and VtR both effectively negated the rise in the serum IL-7 level (Table 1).

Additionally, OVX mice had significantly greater body weight but lower uterine weight than SHAM control at the end of the study (Figure 2). The atrophy of the uterus was negated by 17*β*-estradiol treatment evidenced by dramatically increased uterine weight as compared to OVX. With the data on VtR showing it could not restore the atrophic uterus, we suggest it prevents bone loss major by modulating osteoblast and osteoclast activities, resulting in the regulation of the bone turnover rate rather than influencing the estrogen level.

Bone marrow cells have long been recognized as the source of osteoprogenitor cells [37]. Thus, we applied a culture model of primary bone marrow cells to evaluate the effect of the three major components contained in VtR on the differentiation of osteoblasts (Table 2). Intriguingly, our observations of ALP activity and mineralization suggested that (−)-vitisin B and ampelopsin C possessed higher efficacy in promoting osteoblast differentiation. Cell viability assay found no significant difference between the control and treated groups, suggesting that (−)-vitisin B and ampelopsin C do not influence the proliferation of stromal cells *in vitro*.

Osteoclasts are generated from hematopoietic cells of the monocyte/macrophage lineage [38]. Several lines of evidence have indicated that two key molecules, M-CSF and RANKL, are essential and sufficient to promote osteoclastogenesis [39]. We used this osteoclast differentiation system to examine the efficacy of VtR components and found that (+)-vitisin A under nontoxic concentration ranges most potently suppressed mature MNC formation and bone resorption in a dose-dependent manner (Figure 6(b)). Therefore, (+)-vitisin A could be used as a marker component for anti-osteoporosis therapies involving VtR. Antiresorptive agents are commonly used to treat patients with osteoporosis. When additional increases in bone mass are required to further reduce fracture risk in these patients, bone anabolic agents may be considered as add-on or replacement therapy. Based on the findings that (−)-vitisin B, ampelopsin C, and (+)-vitisin A could reciprocally regulate osteoblastogenesis (the former two) and osteoclastogenesis (the last one), we suggested that VtR, a special ingredient enriched the above three oligostilbees, holds promise as a novel therapeutic drug for the treatment of osteoporosis.

In conclusion, except for its known anti-inflammatory activities, the current study indicates *V. thunbergii* has the potential to protect bone integrity *in vivo*. That is, treatment with VtR at two weeks after ovariectomy significantly attenuated the dramatic decrease in BMD and deterioration in trabecular bone architecture after ovariectomy. The possible mechanism was through upregulation of bone formation and reciprocal downregulation of bone resorption attributed to its diverse constituents. Moreover, this is the first study proving VtR could be a promising alternative to current therapeutic agents for managing of postmenopausal bone loss, although the specific mechanism of the osteoprotective effects of (+)-vitisin A, (−)-vitisin B, and ampelopsin C needs to be clarified.

## Figures and Tables

**Table 1:** Effects of VtR on the serum levels of biochemical markers in OVX mice.

Groups	Osteocalcin (ng/mL)	CTx (ng/mL)	RANKL/OPG (ratio)	IL-7 (pg/mL)
SHAM	17.1 ± 1.5	16.4 ± 0.4	0.31 ± 0.02	0.32 ± 0.05
OVX	23.7 ± 1.4^#^	19.8 ± 0.5^##^	0.83 ± 0.04^##^	1.67 ± 0.13^##^
OVX-VtR-50	22.5 ± 2.2	18.6 ± 0.6	0.88 ± 0.08	1.53 ± 0.28
OVX-VtR-75	27.6 ± 1.7*	16.9 ± 0.7*	0.72 ± 0.03*	0.52 ± 0.19**
OVX-VtR-100	29.3 ± 2.0**	16.3 ± 0.9**	0.42 ± 0.06**	0.39 ± 0.12***
OVX-E	19.4 ± 2.6*	16.4 ± 0.5	0.38 ± 0.07**	0.27 ± 0.08***

Serum levels of biochemical markers in sham-operated mice (SHAM), ovariectomized mice administrated with vehicle (OVX), different dosages (50, 75, and 100 mg/kg) of VtR (OVX-VtR-50, -VtR-75, and -VtR-100), or 17*β*-estradiol (OVX-E) for 8 weeks. The values represent the means ± S.E.M. ^#^ *P* < 0.05 and ^##^ *P* < 0.01, versus sham control; *P < 0.05, **P < 0.01, and ***P < 0.001, versus OVX. *n* = 8–10 samples per group.

**Table 2:** Effects of oligostilbenes isolated from *Vitis thunbergii* and resveratrol on the differentiation and cell viability of primary cultured osteoblasts and osteoclasts, respectively.

Tested compound (20 *μ*M)	Osteoblasts		Osteoclasts	
	ALP activity (ratio)	Cell viability (%)	TRAP activity (ratio)	Cell viability (%)
Basal culture medium	0.42 ± 0.21**	—	0.27 ± 0.09***	—
Control (differentiation medium)	1	100	1	100
(+)-Vitisin-A	1.62 ± 0.22*	103.1 ± 3.5	0.33 ± 0.15***	103.2 ± 2.8
(−)-Vitisin-B	3.39 ± 0.47***	98.2 ± 2.7	0.71 ± 0.04	98.5 ± 4.3
Ampelopsin C	2.75 ± 0.38**	104.5 ± 1.9	0.65 ± 0.12*	97.8 ± 2.9
Resveratrol	1.35 ± 0.21	71.3 ± 2.6***	0.78 ± 0.19	69.7 ± 3.1***

Osteoblast differentiation was measured by culture cells in differentiation medium with or without tested drugs treatment and ALP activity was analyzed as described in Materials and Methods. ALP activity measured in differentiation medium alone was defined as the control. Osteoclast differentiation was measured by culture cells in differentiation medium with or without tested drugs treatment and TRAP activity was analyzed as described in Materials and Methods. TRAP activity measured in differentiation medium alone was defined as the control. Data are mean ± S.E.M. All results were expressed as relative ratio to the control. To preclude the possibility that the attenuation in ALP activity or TRAP activity was due to cytotoxicity, cell viability was measured by MTT assay. *P < 0.05, **P < 0.01, and ***P < 0.001, as compared with the control.

## Abbreviations

ALP: Alkaline phosphatase
CTx: C-Terminal telopeptides of type I collagen
IL: Interleukin
M-CSF: Macrophagecolony stimulating factor
*μ*-CT: Micro-computed tomography
OCN: Osteocalcin
OPG: Osteoprotegerin
RANCL: Receptor activator of NF-*κ*B ligand
TRAP: Tartrate-resistant acid phosphatase.
